# Electroacupuncture Ameliorates Cerebral Ischemia-Reperfusion Injury by Regulation of Autophagy and Apoptosis

**DOI:** 10.1155/2016/7297425

**Published:** 2016-10-09

**Authors:** Shi Shu, Chun-Ming Li, Yan-Li You, Xiao-Lu Qian, Shuang Zhou, Chang-quan Ling

**Affiliations:** Changhai Hospital of Traditional Chinese Medicine, Second Military Medical University, No. 168, Changhai Road, Yangpu District, Shanghai 200433, China

## Abstract

*Background*. The therapeutic mechanisms of cerebral ischemia treatment by acupuncture are yet not well addressed.* Objective*. We investigated the effects of electroacupuncture (EA) at GV26 observing the expression of autophagy-related proteins Beclin-1 and LC3B and proportion of apoptotic cells and Bcl-2 positive cells in MCAO/R model rats.* Methods*. Sprague-Dawley (SD) male rats were randomly assigned to 7 groups: model groups (M6h, M24h, and M72h), EA treatment groups (T6h, T24h, and T72h), and sham operation group (S). Neurological deficit and cerebral infarction volume were measured to assess the improvement effect, while the expression of Beclin-1 and LC3B and proportion of Tunel-positive and Bcl-2 positive cells were examined to explore EA effect on autophagy and apoptosis.* Results*. EA significantly decreased neurological deficit scores and the volume of cerebral infarction. Beclin-1 was significantly decreased in T24h, while LC3B-II/LC3B-I ratio markedly reduced in 6th hour. EA groups markedly reduced the number of Tunel positive cells, especially in T24h. Meanwhile, the number of Bcl-2 positive cells obviously increased after EA treatment, especially in T6h and T24h.* Conclusions*. The alleviation of inadequate autophagy and apoptosis may be a key mechanism involved in the reflex regulation of EA at GV26 to treat cerebral ischemia.

## 1. Introduction

Cerebral ischemia is one of the most devastating types of stroke, which causes high rates of mortality, morbidity, and disability. In China, acupuncture has been used in the treatment of stroke patients for more than 2000 years and GV26 (Shuigou acupoint) is viewed as a classic acupoint in treating stroke in the textbook of Traditional Chinese Medicine [1, 2]. The evidence from clinical study and rats experiment have suggested that EA at GV26 may produce significant benefits for cerebral ischemia injury [3, 4]. The possible mechanisms associated with the therapeutic effects of EA on cerebral ischemia include the influence on cell apoptosis. Meanwhile, autophagy is considered as a new therapeutic target for stroke nowadays and the process by which a membrane engulfs organelles and cytosolic macromolecules to form an autophagosome, with the engulfed materials being delivered to the lysosome for degradation [5–7]. There is no unified theory as to the role autophagy plays in cerebral ischemia and whether activation of autophagy increases or decreases the rate of neuronal death is still under debate. Many studies have suggested that induced autophagy may be a promising neuroprotective strategy in cerebral ischemia, while others considered that either the absence of autophagy or inadequate autophagy may be a key step of cell death [8–11]. However, how does acupuncture work on autophagy in cerebral ischemia? Does activation of autophagy in cerebral ischemia protect neurons from death, or does alleviation of inadequate autophagy decrease cell death? The exact therapeutic mechanisms are yet not well addressed. In this paper, we try to prove the existence of EA effect on autophagy and apoptosis in cerebral ischemia and pose a hypothesis of its possible correlation between autophagy and apoptosis in EA treatment.

## 2. Methods and Experimental Procedure

### 2.1. Experimental Design

The study was divided into two parts: (1) to observe the improvement effect on nerve function damage evaluation and cerebral infarction volume after EA on MCAO/R rats in the following 6th, 24th, and 72nd hour, where sham operation and model without EA were chosen as the control, and (2) to investigate the expression of Beclin-1 and LC3B and proportion of Tunel-positive and Bcl-2 positive cells, trying to prove that EA treatment has beneficial effect by regulation of autophagy and apoptosis.

### 2.2. Animals Preparation

Totally, 210 adult Sprague-Dawley (SD) male rats, weighing from 220 to 250 g, provided by Animal Center, SMMU, were used in the experiment. 140 rats were used in part 1 of the experiment and 70 rats in part 2. All the rats received humane care according to the criteria outlined in the* Guide for the Care and Use of Laboratory Animals* published by the National Institute of Health (NIH publication 86–23 revised 1985). The rats were housed under specific pathogen-free (SPF) controlled conditions with a 12 h light-dark cycle at 25 ± 2°C and with food and water made available before and after surgery.

### 2.3. Experimental Groups

Rats were randomly assigned to the following 7 groups: model groups of the following 6th, 24th, and 72nd hour (M6 h, M24 h, and M72 h), EA treatment groups of the following 6th, 24th, and 72nd hour (T6 h, T24 h, and T72 h), and sham group (S), which underwent the same operation without cerebral artery occlusion.

### 2.4. Surgical Model

Rats were anesthetized with 10% chloral hydrate (400 mg/kg). The left common carotid artery (CCA), external carotid artery (ECA), and internal carotid artery (ICA) were exposed. A 30 mm long monofilament nylon suture was inserted from the right CCA to the internal carotid artery (ICA) through the stump of the ECA and then advanced to block the origin of the right middle cerebral artery (MCA). Rats were anaesthetized 2 h after MCAO, and reperfusion was achieved by withdrawal of the suture from the ECA. Animals in the sham group were subjected to the same surgical procedures but without insertion of nylon suture into the MCA [12]. The mortality of MCAO/R model is 18%. The criteria of a successful MCAO/R model are as follows: (1) tail suspension test (lift mouse tail one meter above the ground): rats that cannot stretch ischemia contralateral forelimb independently; (2) rats that consistently reduced resistance to lateral push toward the paretic side; (3) rats that circled toward the paretic side consistently. Any of the above situations means occlusion of MCA successfully [13].

### 2.5. EA Treatment

Rats were anaesthetized and immobilized on the wooden board. Two stainless acupuncture needles (0.25 mm outer diameter) were subcutaneously inserted 2-3 mm into the GV26 acupoints and right sulcus auriculae posterior and were left for 30 min. The electrical stimulation was made by a medical EA apparatus (Model G6805-2, Shanghai, China), connecting the above two needles with the frequency of 2 and 20 Hz, alternatively, and intensity strong enough to only elicit slight twitches of the orofacial areas. GV26 are located in the depression below the septum, 2/3 of the way up [14], while right sulcus auriculae posterior was an indifferent electrode located in the posterior of right auriculae.

### 2.6. Neurological Scores

The neurological scores were measured after 6 h, 24 h, and 72 h reperfusion following 2 h ischemia which were based on the scoring system of Bederson et al. (*n* = 15 from each group). The score given to each rat at the completion of the evaluation was divided into 4 basic grades, from 0 to 3. The minimum neurological score grade 0 refers to “no observable deficit”; grade 1 refers to “forelimb flexion”; grade 2 refers to “decreased resistance to lateral push (and forelimb flexion) without circling”; and grade 3 refers to “the same behavior as grade 2 with circling” [13].

### 2.7. Cerebral Infarction Volume

The rat brains were dissected coronally and sliced into 2 mm thickness. 2,3,5-Triphenyltetrazolium chloride (TTC, Xinong Inc., Beijing, China) was used to stain these coronal brain sections for 30 min and then fixed with 10% paraformaldehyde, in order to distinguish the cerebral infract of the affected brain tissue. The red and white brain regions of the TTC-stained brains were separated obviously. The sections were scanned and analyzed by using computerized image analysis software (Image-Pro-Plus). Infarct volume was analyzed using 5 slices of 3 mm coronal sections from each brain and calculated with the following formula: infarct volume (%) = (contralateral hemisphere volume − surgery side without infarct volume)/contralateral hemisphere volume × 100% [15, 16].

### 2.8. Western Blot

The ischemic cortex and nonischemic controls from each group were dissected for western blot (*n* = 4 per group). Tissues were homogenized in ice-cold lysis buffer. The protein content was determined by Bio-Rad protein assay. Equal amounts of protein per lane (50 *μ*g) were loaded onto an 8~12% polyacrylamide gel and separated by electrophoresis at 80 V for 30 min and then 120 V for 1.5 h. Proteins were then transferred to nitrocellulose at 250 V for 1 h and the membrane was blocked with 5% nonfat dry milk/0.5% Tween-20 in Tris-buffered saline for 1 h. The nitrocellulose was then incubated with different antibodies overnight at 4°C: rabbit anti-Beclin (D40C5) 1 : 1000, and rabbit anti-LC-3 (D11) 1 : 1000 (Santa Cruz Inc., Santa Cruz, CA, USA). The membrane was treated with horseradish peroxidase-conjugated secondary antibody for 60 min at room temperature. Immunoblots were probed and then exposed to X-ray film. The X-ray films were scanned and the optical density was determined by Bio-Rad Image analysis. As an internal control, the same nitrocellulose was incubated with an antibody specifically for *β*-actin (Santa Cruz, 1 : 1000) after being stripped.

### 2.9. Tunel Assay

Tunel staining was performed with Roche in situ cell death detection kit (cat. number 11684817910). After fixing, the 5 *μ*m thick paraffin-embedded sections were deparaffinized and rehydrated and incubated with proteinase K (10 *μ*g/mL) for 15 min. After washing 3 times in phosphate-buffered saline (PBS), sections were incubated with reaction buffer containing enzyme at 37°C for 30 min in the dark. DAPI (4′,6-diamidino-2-phenylindole) staining was performed to visualize all the nuclei and then apoptotic cells (stained with brown fluorescence) were detected randomly using a confocal fluorescence microscope. The positive cells and total cells were counted from five random fields in each slice under light microscopy (×200); then, the average positive cells of each sample were obtained by calculating the average of the three slices. The result was expressed as a ratio of the positive cell number to the total cell number (*n* = 3 for each group) [17].

### 2.10. Immunohistochemistry

5 *μ*m thick brain sections were immersed in 3% hydrogen peroxide (H_2_O_2_) and then incubated with Bcl-2 rabbit polyclonal antibodies (1 : 100; Santa Cruz Biotechnology, CA, USA) in 0.01 mol/L phosphate-buffered saline (PBS) over night at 4°C. The secondary antibodies were applied for 50 min at room temperature. After repeated washing in PBS, the slides were mounted and DAB kit (DAKO, Denmark) was used for staining. After washing and dehydration, the positive cells with claybank or pale yellow fluorescence were evaluated using a fluorescence microscope with appropriate filters (×200). The counting method was performed as described above in Tunel assay. The result was expressed as a ratio of the positive cell number to the total cell number (*n* = 3 for each group).

### 2.11. Statistical Analysis

All data were processed by SPSS21.0, and results were expressed as mean ± standard deviation. The statistical significance of differences among different groups was determined with a 95% confidence interval by the ANOVA test for normally distributed data. Significant differences between groups at each time point were assessed by LSD *t*-test. *P* < 0.05 was considered to be statistically significant.

## 3. Results

### 3.1. Assessment of Neurological Function

**Figure 1:**
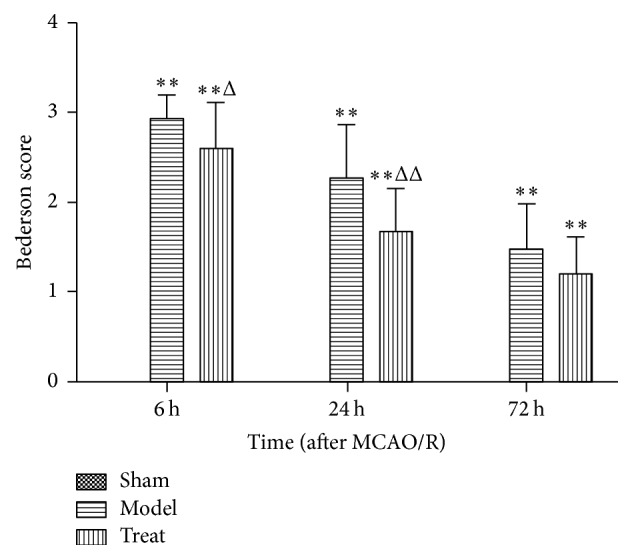
Assessment of neurological function with Bederson score on postoperative 6 h, 24 h, and 72 h in sham groups, model groups, and EA treatment groups (*n* = 15 from each group).   ^*∗∗*^ *P* < 0.01 versus sham group; ^Δ^ *P* < 0.05 and ^ΔΔ^ *P* < 0.01 versus model group in the corresponding time.

Sham group rats did not show neurobehavioral impairments signs; Bederson scores are 0; the Bederson scores of model groups rats after ICH are higher: about 2.93 ± 0.26, 2.57 ± 0.59, and 1.47 ± 0.52 in M6 h, M24 h, and M72 h; the difference compared with sham group was statistically significant (*P* < 0.01). Compared with model group, Bederson scores of EA group rats decreased: about 2.60 ± 0.51, 1.67 ± 0.49, and 1.20 ± 0.41 in M6 h, M24 h, and M72 h and the difference was statistically significant (*P* < 0.05) (Figure 1).

### 3.2. Cerebral Infarction Volume

**Figure 2:**
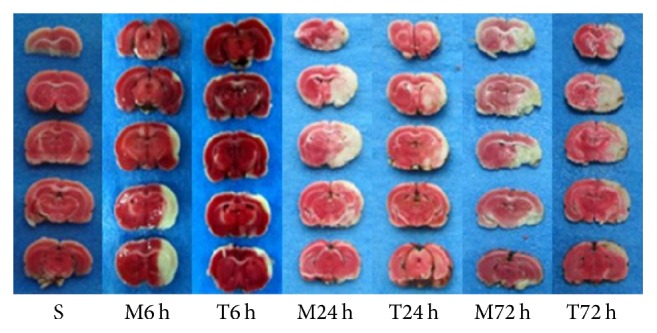
TTC staining images of rats in each group. The seven groups are model groups of the following 6th, 24th, and 72nd hour (M6 h, M24 h, and M72 h), EA treatment groups of the following 6th, 24th, and 72nd hour (T6 h, T24 h, and T72 h), and sham operation group (S).

**Figure 3:**
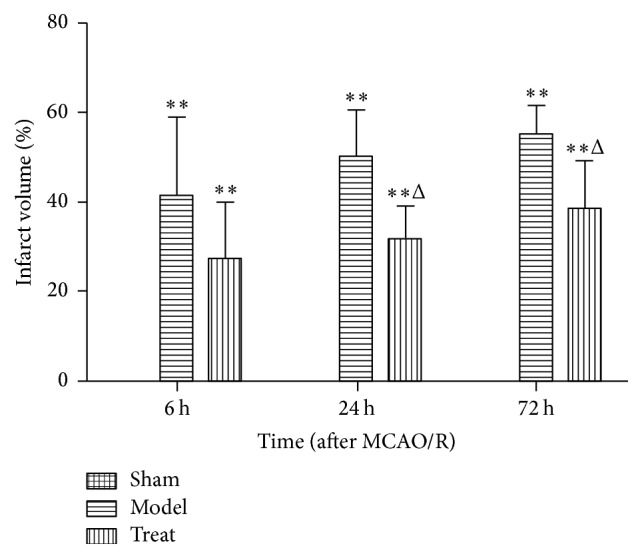
Cerebral infarction volume of MCAO/R rats in each group (*n* = 5 from each group).   ^*∗∗*^ *P* < 0.01 versus sham group; ^Δ^ *P* < 0.05 versus model group in the corresponding time.

As illustrated in figure of TTC staining images, rat brain in model and EA groups appear as obvious white infarction area (Figure 2). Compared with model group, rat infarction volume of EA groups in 24 h and 72 h was significantly decreased (32.00 ± 7.11 versus 50.20 ± 10.52; 38.60 ± 10.80 versus 55.00 ± 6.74; *P* < 0.05), while there is no significant difference in 6 h groups (*P* > 0.05) (Figure 3).

### 3.3. Effect on the Expression of Autophagy-Related Proteins

**Figure 4:**
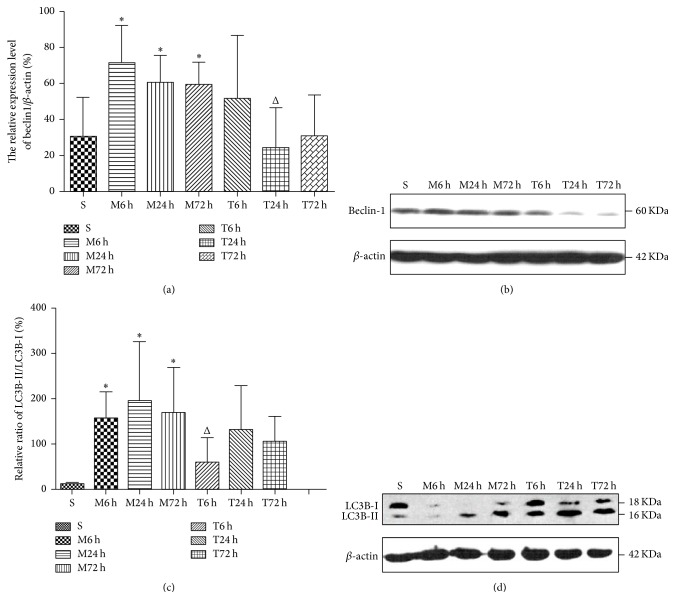
Relative quantity of Beclin and LC3B protein expression in groups of rats' cerebral cortex by western blot (*n* = 4 from each group). Representative western blotting images are shown in panels (b) and (d). The quantification results are presented in panels (a) (relative to *β*-actinin internal control) and (c) (the ratio of LC3B-II/LC3B-I). ^*∗*^ *P* < 0.05 versus sham group; ^Δ^ *P* < 0.05 versus model group in the corresponding time.

Rats in sham group only had a little Beclin protein expression. Compared with the sham group, Beclin protein in cerebral cortex of model group rats increased: about 71.55 ± 20.87, 60.46 ± 15.19, and 59.13 ± 12.86 in M6 h, M24 h, and M72 h; the difference was statistically significant (*P* < 0.05), and 1~3 days after operation the expression was still at a high level. However, the expression of Beclin protein in EA group rats obviously reduced: about 51.28 ± 35.38, 24.50 ± 22.07, and 30.61 ± 23.08 in T6 h, T24 h, and T72 h, and there is a statistically significant difference (*P* < 0.05) between EA group and model group after 24 hours (Figure 4). Meanwhile, LC3B protein levels markedly increased in model groups, compared with sham groups: 157.27 ± 57.83, 194.86 ± 131.55, and 168.84 ± 100.18, *P* < 0.05. LC3B protein of EA group rats obviously reduced: about 55.28 ± 55.54, 130.98 ± 98.31, and 105.32 ± 55.96 in T6 h, T24 h, and T72 h, and there is a statistically significant difference between T6 h versus M6 h (*P* < 0.05).

### 3.4. Neuronal Apoptosis

**Figure 5:**
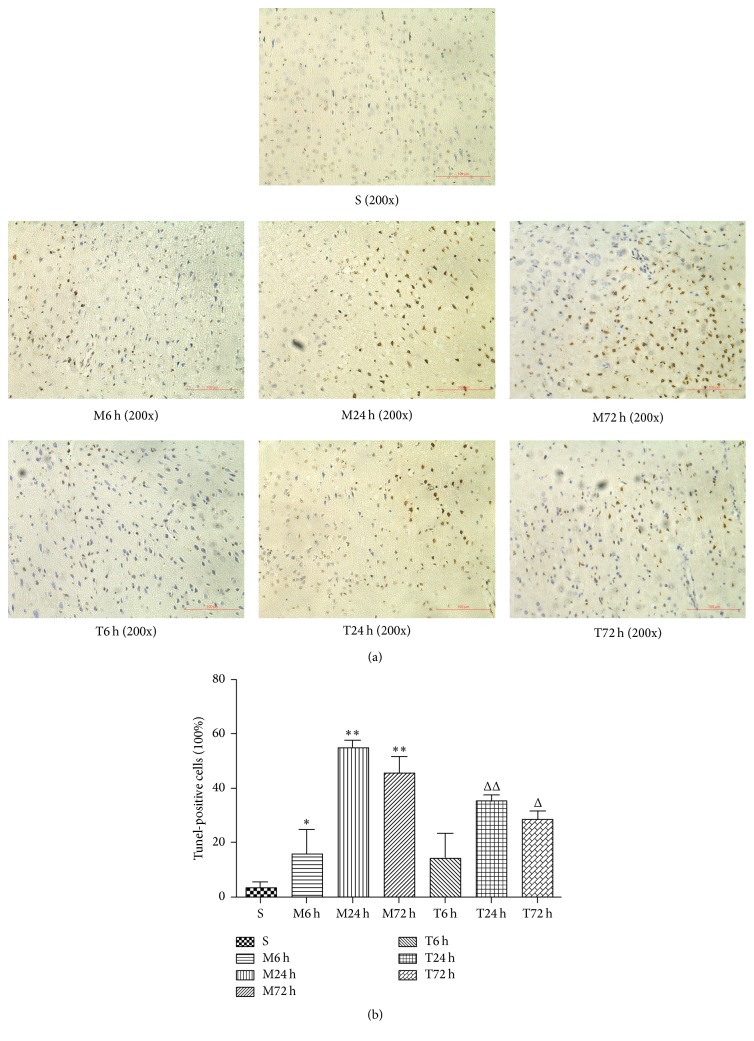
Photomicrograph images (a) and proportion of Tunel-positive cells (%) (b) in the cerebral cortex (*n* = 3 from each group). ^*∗*^ *P* < 0.05 and ^*∗∗*^ *P* < 0.01 versus sham group; ^Δ^ *P* < 0.05 and ^ΔΔ^ *P* < 0.01 versus model group in the corresponding time.

Tunel staining is a major marker of cell apoptosis.  Figure 5 shows the apoptotic changes of ischemia-reperfusion injury and EA treatment in the cerebral cortex. Few apoptotic cells exist in the sham group (2.98 ± 2.34%). In the model groups, greater proportion of Tunel-positive cells and condensation of nuclear fragments markedly increase, about 15.33 ± 9.29%, 54.73 ± 2.90%, *P* < 0.01, and 45.33 ± 6.11% in M6 h, M24 h, and M72 h. In contrast, EA treatment reduced the number of apoptotic cells obviously compared with model groups, respectively, especially in T24 h group (about 34.67 ± 2.52%, *P* < 0.01, versus M24 h group) (Figure 5).

### 3.5. Immunohistochemistry for Bcl-2

**Figure 6:**
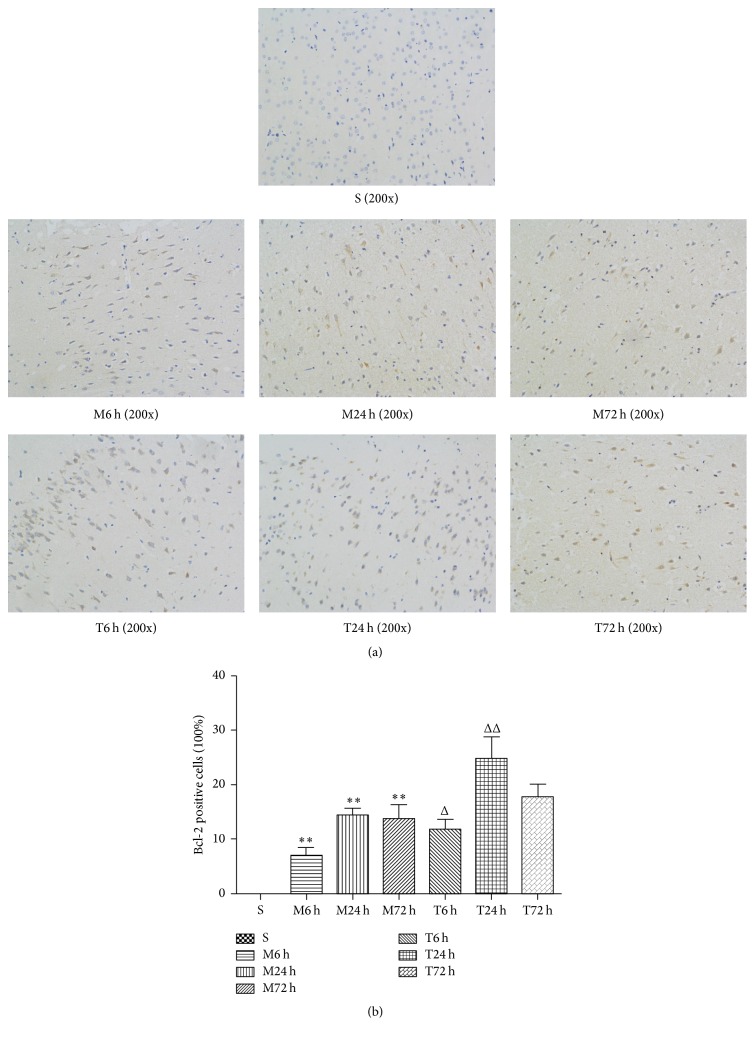
Photomicrograph images (a) and proportion of Bcl-2 positive cells (%) (b) in the cerebral cortex (*n* = 3 from each group).   ^*∗∗*^ *P* < 0.01 versus sham group; ^Δ^ *P* < 0.05 and ^ΔΔ^ *P* < 0.01 versus model group in the corresponding time.

The positive cells of Bcl-2 under fluorescence microscope appear as claybank or pale yellow fluorescence. As shown in Figure 6, there are no positive cells in sham group, while greater proportion of Bcl-2 positive cells markedly increase in three-time model groups, about 6.87 ± 1.59%, 14.49 ± 1.12%, and 13.74 ± 2.60%. Meanwhile, the number of Bcl-2 positive cells obviously increases after EA treatment compared with model groups, respectively, especially in T6 h group (11.83 ± 1.78%, *P* < 0.05, versus M6 h group) and T24 h group (24.75 ± 4.13%, *P* < 0.01, versus M24 h group) (Figure 6).

## 4. Discussion

It is well known that stroke is one of the most common diseases for which acupuncture treatment is recommended, according to the World Health Organization [18, 19]. Approximately 70% of stroke cases in China are due to primary cerebral ischemia resulting in infarction. Currently, acupuncture is widely accepted by Chinese people and increasingly patients in western countries [20–22]. Some reports have also confirmed that EA as a treatment method can improve the cerebral IR injury [3, 4, 17, 23, 24]. Neurological scores and cerebral infarction volume have been widely used as the effective evaluation criterion for cerebral injury. In this study, both neurological scores and cerebral infarction volume show brain recovery in EA treatment groups rats was marked, which supports the suggestion that EA at GV26 indeed produces significant benefits for cerebral ischemia.

There are three different types of cell death, including apoptosis (Type I), autophagy (Type II), and necrosis (Type III). In ischemic neuronal death, apoptosis (“self-killing”) and autophagy (“self-eating”) influence stroke development and progression and the functional relationship between apoptosis and autophagy is complex under ischemic circumstances [25, 26]. Little is known about the functional significance of this interaction. Autophagy is a double-edged sword which makes an alternative cell death pathway, whereas in other cellular conditions, it could avoid cell death with a stress adaptation. Apoptosis and autophagy are both triggered by common upstream signals. Sometimes these may cause combined responses of autophagy and apoptosis; in other instances, the responses play in a mutually exclusive manner [5].

In the crosstalk of apoptosis and autophagy, the antiapoptotic protein Bcl-2 plays a central role and regulates autophagy process by interacting with autophagy protein, Beclin. Beclin-1 interacts with Bcl-2 family proteins through its Bcl-2-homology-3 (BH3) amphipathic alpha-helix (amino acids 112–123). Some other studies have shown that the complex of Beclin-1 and other antiapoptotic factors inhibits Beclin-1-dependent autophagy. On the contrary, proapoptotic BH3-only proteins induce autophagy by freeing Beclin from the complex. These results further substantiate the notion that Bcl-2 also controls autophagy [27, 28].

Is the effect of EA treatment on cerebral IR injury correlated with autophagy of neurons? The present study examined expression of autophagy-related proteins Beclin-1 and LC3B by western blot. After cerebral IR injury, both Beclin-1 and LC3B proteins increased in cerebral cortex, which indicated that autophagy was activated. However, once the EA intervention was given, Beclin-1 and LC3B proteins in all the phases of EA group were decreased significantly. In that case, this indicated that the effect of EA treatment may alleviate inadequate autophagy to avoid cell death. This is consistent with the result of our previous study that EA could reduce the formation of autophagosome with transmission electron microscopy. Moreover, IR greatly increased apoptosis in the cortex and the results of tunnel staining and proportion of Bcl-2 positive cells also suggest that EA treatment may reduce neuronal apoptosis, which is combined with the progress of neural autophagy.

## 5. Conclusion

To sum up, the alleviation of inadequate autophagy and apoptosis may be a key mechanism involved in the reflex regulation of EA at GV26 to treat cerebral ischemia. In this process of EA treatment, the two responses of autophagy and apoptosis may be in a mutually synergistic manner inhibiting Beclin-dependent autophagy by upregulation of antiapoptotic protein Bcl-2 which maybe involved in this process. However, the above conclusion still needs further more concrete experimental evidence, which our study will focus on in future.
